# Abdominal skin tensile strength in aesthetic and massive weight loss patients and its role in ventral hernia repair

**DOI:** 10.1186/s12893-019-0523-7

**Published:** 2019-06-26

**Authors:** Guilherme Barreiro, Vinícius S. de Lima, Leandro T. Cavazzola

**Affiliations:** 1 Hospital de Clínicas de Porto Alegre, Universidade Federal do Rio Grande do Sul, Porto Alegre, Brazil; 2 Hospital Conceição, Porto Alegre, Brazil; 3 Case Western Reserve University, Cleveland, OH USA; 4 Universidade Federal do Rio Grande do Sul, Porto Alegre, Brazil; 5 Sinop/MT, Brazil

**Keywords:** Abdominoplasty, Hernia repair, Plastic surgery, Bariatric surgery

## Abstract

**Background:**

Clarifying the biomechanics of abdominal skin could lead to different uses for this tissue such as the ventral repair of hernias in patients with excess skin and incisional hernias. The objective of this study was to compare the maximum tensile strength of abdominal skin to commercial meshes and to verify whether or not it varies between aesthetic patients and massive weight-loss patients.

**Methods:**

Experimental cross-sectional study. Skin samples sized 32 × 20 mm were taken from 15 abdominoplasties and 10 panniculectomies. The skin specimens were analyzed in vertical and horizontal tensile strength tests. Results were compared between the two groups including their traction directions. Commercial meshes were also tested. The results were analyzed using the Generalized Estimating Equation.

**Results:**

The maximum tensile strength supported vertically by abdominal dermis was 403.5 ± 27.4 N in the abdominoplasty group and 425.9 ± 33.9 N in the panniculectomy group. Horizontally, the values were 596.5 ± 32.2 N and 612.5 ± 43.9 N respectively. The strengths between traction directions were significantly different (*p* < 0.001). There were no differences between the groups with regard to the maximum tensile strength (*p* = 0.472). Tested commercial meshes had the following values: polypropylene 104.6 N, low-weight polypropylene 54.4 N, polytetrafluorethylene (PTFE) 82.2 N, and hydrated porcine small-intestinal submucosa 60.0 N.

**Conclusion:**

In our study, the tensile strength of the tested human abdominal dermis samples, both aesthetic and post-bariatric, was superior to the commercial meshes. Therefore, in selected cases, abdominal dermis could be an alternative tool in abdominal reconstruction during panniculectomies with concomitant hernia repair.

**Electronic supplementary material:**

The online version of this article (10.1186/s12893-019-0523-7) contains supplementary material, which is available to authorized users.

## Background

Incisional hernias are classified within the spectrum of ventral hernias, developing at sites of previous abdominal incisions. For this reason, their incidence is closely linked to the number of primary surgical interventions. With the recent increase in the number of laparotomies and laparoscopies, there has also been a rise in both the incidence of incisional hernias and the absolute costs generated with their treatment [1].

One of the main causative factors for incisional hernias is obesity. Data from the World Health Organization (WHO) indicate that at least 2.8 million persons worldwide die annually due to being overweight [2]. The increase in the incidence and prevalence of this condition, as well as associated morbidity and mortality, have led to a 13% growth in the demand for bariatric surgeries between 2011 and 2013 in the U.S [3, 4]. One of the recent major complications related to this treatment is the development of incisional hernias, which can occur in up to 24% of cases in which laparotomic access is employed [5].

In the treatment of incisional hernias, hernioplasty with the use of meshes, both synthetic and biological, has established itself as the gold standard [6]. The use of prostheses, conceived by Billroth in the nineteenth century, aimed to reinforce the musculoaponeurotic component in contrast to the tendency of organs to insinuate themselves through the abdominal wall. The ideal mesh as proposed by Shankaran would be non-carcinogenic, capable of being sterilized, chemically inert, unlikely to produce a significant immune reaction, resistant to mechanical forces, infection, and visceral adherences, as well as amenable to large-scale production [7]. Unfortunately, that mesh does not yet exist, and currently used materials produce complications that cannot be ignored such as post-operative pain, infection, and recurrences [8].

The use of skin as a mesh in the correction of ventral hernias is a technique developed over 30 years ago; it is a popularly used treatment in European countries such as Germany, Italy, France, and Russia [9–11]. The technique’s description, however, is hard to access because the lion’s share of studies are described in the native language, which limits their impact in the global literature. Kama and collaborators demonstrated the proper performance of dermal autografts for correction of ventral hernias in an experimental study in an animal model [12]. In another relevant article, Korenkov and collaborators compared abdominal reconstructions in patients with simple and complex hernias with simple sutures to the use of autologous or alloplastic materials; they found no significant differences between the meshes and the autograft [13]. Existing comparative studies in the global literature provide evidence of autologous dermal mesh grafting as a tool that is, at a minimum, not inferior to commercial meshes in terms of clinical outcomes [12, 13]. Since it is an autogenous material, it is also possible that the autograft would have lower rates of complications and lower costs.

The objective of the present study was to verify the maximum tensile strength of abdominal skin and compare it to the major commercially available meshes. Aesthetic and post-bariatric patients were assessed to allow comparison between the groups, and different directions of skin traction were analyzed. The working hypothesis of the research group was that the maximum tensile strength of the abdominal skin does not differ between aesthetic and post-bariatric patients, and that both are superior to commercial meshes.

## Methods

This was an experimental cross-sectional study that evaluated as an outcome the maximum tensile strength of abdominal skin, in Newtons (N), of 25 patients who underwent abdominoplasties, 15 of which were aesthetic and 10 post-bariatric. Owing to the large influence of the measurement protocol in the parameter under evaluation, the choice was made not to use data from the literature to calculate the sample size, which was arbitrarily defined. The patients were enrolled sequentially at a Brazilian public hospital during the period from May to November 2015; patients who had previously undergone abdominoplasties, or presented with collagen diseases or any comorbidity that could significantly alter skin biomechanics were excluded.

The clinical histories of the patients were collected and included comorbidities, tobacco usage profile, and obstetric history. Anthropometric data were recorded using a graduated tape measure, with the goal of comparing the biotypes of the two groups. The abdominal circumference was defined as the smallest circumference measured between the end of the rib cage and the iliac crest. The xiphoid-genital distance was defined as the distance in a straight line between the xiphoid process and the pudendal cleft. The xiphoid-umbilical distance, like the umbilical-genital distance, considered skinfolds in such a way that, in some cases, their sum is a value greater than the xiphoid-genital distance. The angle of dorsiflexion of the surgical table was measured at the completion of the surgery using a digital goniometer (Everise Medical, Jiangsu, China). The dimensions of the resected specimen were also measured using graduated metric tape.

**Fig. 1:**
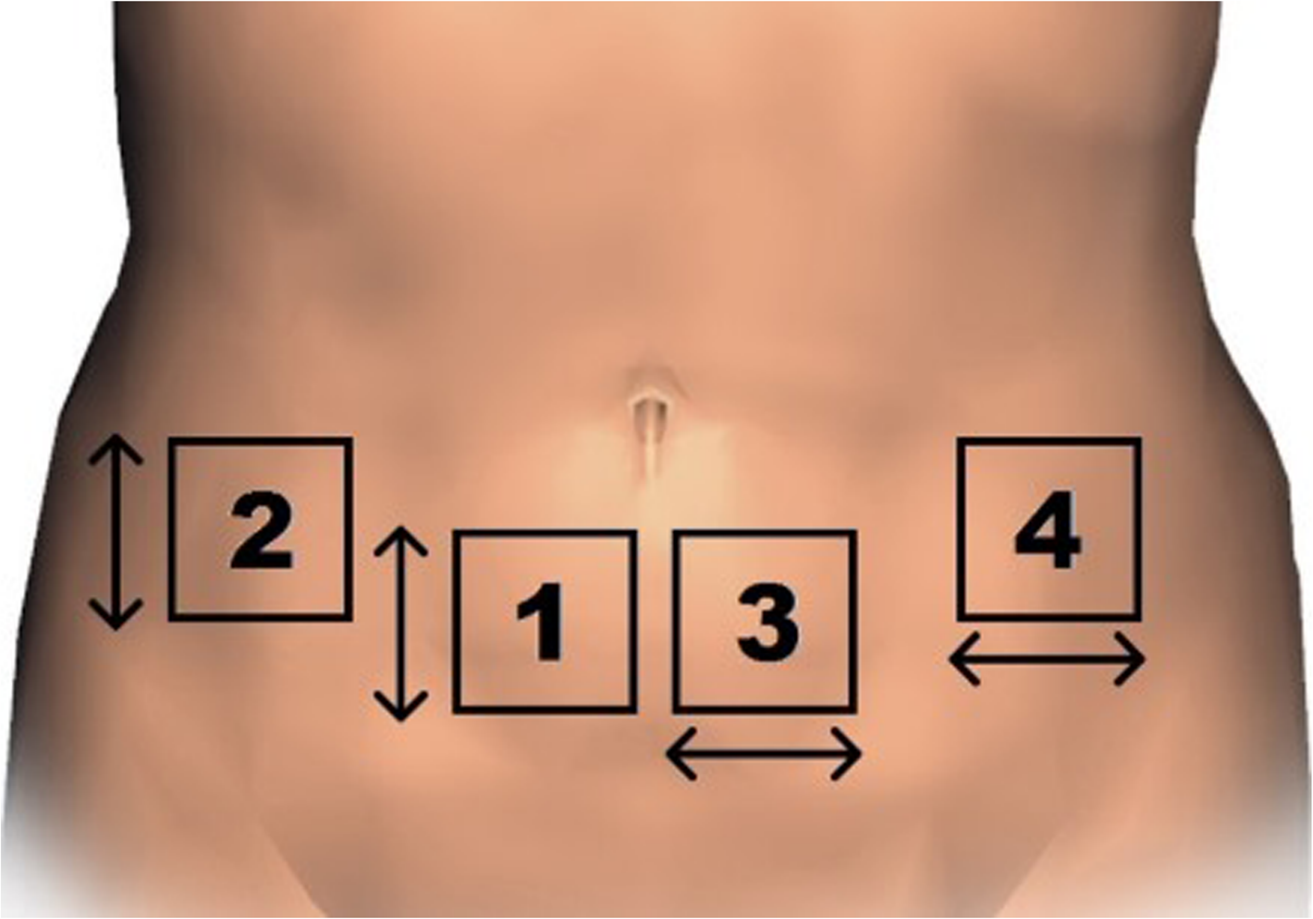
Samples positions

**Fig. 2:**
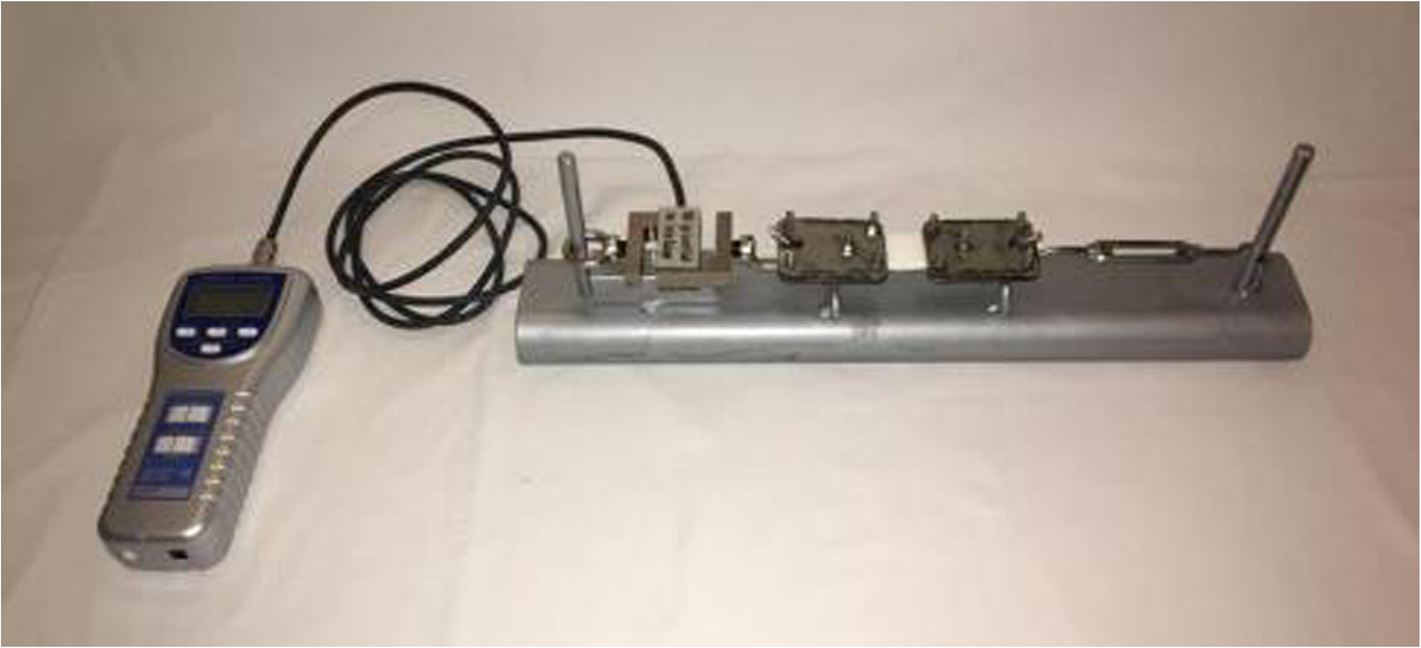
System of clamps and screws used for graduated distension of the skin

Of the dermal fat specimens resected during the surgery, four fragments measuring 32 × 20 mm (mm) were extracted, two for testing in the vertical direction relative to the main abdominal axis (one from the medial portion of the flap and others from the lateral portion), and two for testing in the horizontal direction, in the same position (Fig. 1) [14]. Samples of total skin were isolated from the subcutaneous tissue and submitted to a protocol defined by the research team. A system of clamps and screws was used for graduated distension of the skin with simultaneous measurement of the force produced by a dynamometer (Instrutherm®, DD-300 model) calibrated to the *peak hold* function (Fig. 2). The traction speed was defined as 5% extension of skin per second and kept constant until the rupture of the specimen.

For purposes of comparing human skin with commercial meshes, one sample of the same size from four different meshes was submitted to the identical protocol. Meshes made of high- and low-density polypropylene (Ethicon, Sommerville, NJ), polytetrafluoroethylene (W.L. Gore&Associates, Flagstaff, AZ, USA) and hydrated porcine small intestinal submucosa (Cook Medical, Bloomington, IN, USA) were evaluated. The same traction speed was observed until total rupture of the sample being tested.

For the statistical analysis of the outcomes, the Generalized Estimating Equation (GEE) model was used, by which it was possible to identify interactions between the groups of aesthetic and post-bariatric patients and between different collection topographies. The calculation of the study power to detect non-inferiority was one-tailed and performed with the software WINPEPI®. All the other tests were two-tailed, with *p* < 0.05 defined as significant. The demographic and biometric characteristics were compared between the groups using the t-test, chi-squared distribution, and the Mann-Whitney U test. The data are presented as mean ± standard deviation (SD) or median (interquartile range). The project was approved by the Hospital de Clinicas de Porto Alegre Ethics Committee under CAAE 41787915.1.1001.5327, and all patients signed a consent form to participate in the research and to publish their cases. The Strengthening the Reporting of Observational Studies in Epidemiology (STROBE) checklist was used for the study report and its results [15].

## Results

### Clinical features

Fifteen aesthetic patients and 10 patients with massive weight loss were assessed. All of the analyzed patients were female. The mean age of the aesthetic patients was 37.2 years (range: 22 to 53 years) and that of the post-bariatric patients was 45.9 years (range: 30 to 62 years), a significant difference (*p* = 0.037). The aesthetic patients suffered less frequently from comorbidities (26.7% vs. 50%), but without statistical significance in the difference between the groups (*p* = 0.397). The number of pregnancies showed no differences (*p* = 0.232). The demographic data are presented in Table 1.

**Table 1:** Demographic Characteristics

	Aesthetic Patients	Post-bariatric Patients	*p*
No.	15	10	
Age (years)			
Mean	37.2	45.9	0.037^*^
Range	22–53	30–62	
Comorbidities (%)			
Yes	26.7	50	0.397^#^
No	73.3	50	
Tobacco use (%)			
Yes	0	0	0.017^#^
No	100	60	
Ex-smoker	0	40	
Pregnancies, median (IQ)	2 (1–3)	3 (1.5–5)	0.232^×^
Mean BMI ± SD	25.7 ± 1.3	29.5 ± 4.7	0.035^*^

^*^t-test; ^#^ chi-squared; ^×^Mann-Whitney *U* test

With respect to the anthropometric data, the abdominal circumference tended to be greater among the patients with massive weight loss (83.8 cm vs. 90.7 cm), but without statistical significance (*p* = 0.166). The body mass index (BMI) of the aesthetic patients was 25.7 ± 1.3 kg/m^2^, significantly less than that of the post-bariatric patients, whose BMI was 29.5 ± 4.7 kg/m^2^ (*p* = 0.035). In addition, the only statistically significant difference was the distance between the navel and the genital region, which was 22 cm for the aesthetic patients and 25 cm for the other group (*p* = 0.002).

The dermal fat flaps resected from post-bariatric patients were significantly larger in their horizontal dimension, averaging 50.5 cm, while the mean in aesthetic patients was 32.4 cm (*p* = 0.002). The values from vertical measurement also tended to be greater, although without being mathematically significant (*p* = 0.095). The anthropometric and surgical data are shown in Table 2.

**Table 2:** Biometric and Surgical Characteristics

	Aesthetic	Post-bariatric	*p*
No.	15	10	
Abdominal circumference mean ± *SD* (cm)	83.8 ± 4.4	90.7 ± 14.0	0.166^*^
Xiphoid-genital distance mean ± *SD* (cm)	36.6 ± 1.9	37.6 ± 4.3	0.536^*^
Xiphoid-umbilical distance mean ± *SD* (cm)	18.4 ± 1.9	19.7 ± 3.4	0.261^*^
Umbilical-genital distance median (IQ) (cm)	22 (18–22)	25 (23.25–27.25)	0.002^×^
Dorsiflexion angle mean ± *SD* (degrees)	154.0 ± 7.4	162.2 ± 13.6	0.106^*^
Craniocaudal size of resection ± SD (cm)	19.0 ± 2.7	21.8 ± 2.1	0.095^*^
Latero-lateral size of resection ± *SD* (cm)	32.4 ± 3.6	50.5 ± 12.9	0.002^*^

^*^t-test; ^×^Mann-Whitney *U* test

### Maximum tensile strength

When the tensile strength of the skin was analyzed, the statistical model used did not detect a difference between the aesthetic and the post-bariatric patients (*p* = 0.472). Considering the sample size and the standard deviations of both groups, the study is able to say that there is no difference greater than 100 N in the post-bariatric patients compared to the aesthetic patients, with a power of 85%. The traction direction greatly influenced the values in both groups, leading to the conclusion that the tensile strength of the skin measured in the vertical direction is significantly different than that measured in the horizontal direction (*p* < 0.001).

**Fig. 3:**
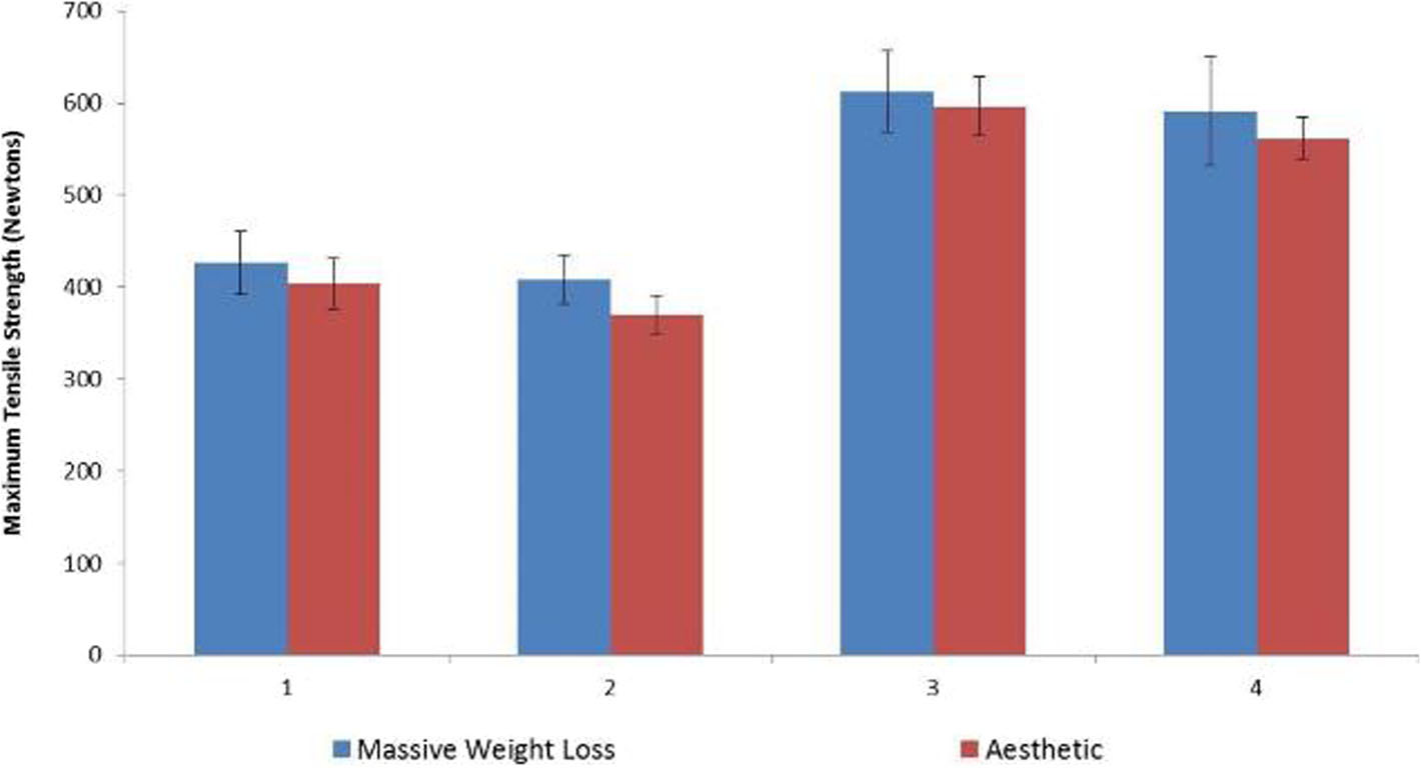
Aesthetic and massive weight loss abdominal skin tensile strengths

Measurement 1 was 425.9 N in the post-bariatric patients and 403.5 N in the aesthetic patients. When the skin from the lateral portion of the abdomen was analyzed, reported by the research team as Measurement 2, there were similar values of 407.1 N and 369.7 N, respectively. When the skin was pulled in the horizontal direction, on average (Measurement 3) the values were 612.5 N in the post-bariatric patients and 596.5 N in the aesthetic patients. In the lateral skin of the abdomen, designated Measurement 4, the latter had values of 561.3 N, while results of the former were 591.3 N (Fig. 3).

When submitted to the same evaluation protocol, the commercial meshes yielded the following maximum tensile strength values: high-density polypropylene: 104.6 N; low-density polypropylene: 54.4 N; polytetrafluoroethylene: 82.2 N; and hydrated porcine small intestinal submucosa: 60.6 N. The values are reported in Table 3.

**Table 3:** Maximum Tensile Strength of Commercial Meshes Tested

Mesh	Strength
High-density polypropylene	104.6 N
Low-density polypropylene	54.4 N
PTFE	82.2 N
Hydrated porcine small-intestinal submucosa	60.6 N

*N* Newtons, *PTFE* polytetrafluorethylene

## Conclusion

With regard to the characteristics of the groups, differences identified in age and BMI were statistically significant between the groups. However, despite the mathematical significance, these differences hardly play a clinically relevant role with respect to skin biomechanics since they have a reduced absolute value (8 years apart and 4 BMI points). The greatest distance from the navel to the genital region presented by the post-bariatric patients was already expected, in view of the suprapubic abdominal crease characteristic of this group, popularly known as the panniculus or “abdominal apron” [16].

Concerning the maximum tensile strength, the statistical analysis of the biomechanical results of our study did not find a significant difference between the skin of post-bariatric and aesthetic patients (Additional file 1). The large standard deviation of the studied variable caused the sample size required for equivalence or non-inferiority studies to be high. However, even with a sample of only 25 patients, statistically we can say—given a beta error of 0.15—that the inferiority of the post-bariatric patients compared to the aesthetic patients, if any, does not exceed 100 N. In the context of the values presented, even if we assume that the abdominal skin of post-bariatric patients has a lower maximum tensile strength, its value would still be greater than that of the commercial meshes.

When the traction directions were compared, it was noted that the skin tissue had a greater resistance to being tested horizontally. This information can have a practical application in providing guidance for the positioning of grafts in corrective surgery for ventral hernias.

### Outlook for the future

The study conducted by Mutlu and collaborators showed maximum tensile strength values of the abdominal skin similar to the values that we found [17]. In a series of 12 cases corrected with autograft, after an average follow-up period of 26 months, there were no recurrences diagnosed by the authors in clinical examination or in radiological study by MRI. However, only patients without a history of metabolic surgery were evaluated, just as a single traction direction was measured during the biomechanical tests.

Obesity is an independent risk factor for the development of ventral hernias [18]. Bariatric surgery, because it is performed in this population profile, ultimately results in a significant number of patients suffering from this pathology. Many of them will undergo reconstructive plastic surgery to improve their quality of life, during which there will be resection of brachial, crural, and abdominal dermal fat tissue, which today is discarded [19, 20].

The reuse of skin tissue as a substrate for abdominal reconstruction could reduce costs, minimize complications, and improve outcomes in the treatment of ventral hernias following bariatric surgeries. For this, however, it is essential to compare the biomechanical properties of the autograft with those of meshes currently in use. Our study used maximum tensile strength as the only outcome, which certainly does not exhaust the comparison of the two materials that is necessary [21]. Properties like elasticity and integration may differ significantly in the long term and were not the subject of the research study presented here.

The main limitation of our study is its design. It is known that, in some cases, experimental studies do not confirm their results in clinical trials and projects with greater methodological rigor. Another possible limitation was the non-de-epithelialization of the samples due to cost reduction issues. Although the epidermis does not significantly contribute to tensile strength, it is possible that its removal could cause a slight change in the abdominal skin tensile strength.

The results demonstrated in this research have opened new possibilities for future studies that approach dermal autograft as a tool for abdominal wall reconstruction in post-bariatric patients. This technique, if its benefits are confirmed, may make the plastic surgeon an important member of the multidisciplinary team assisting these individuals. In addition to its already recognized protagonism in the recovery of body contouring, rehabilitation of the musculoaponeurotic abdominal wall with the use of autologous material would open new horizons for global plastic surgery.

## Additional file

Additional file 1: Aesthetic and post-bariatric data. Two sheets with clinical and numerical data collected from both groups of patients. The vertical and horizontal tensile strength tests are identified as MED1, MED2, MED3 and MED4. (XLSX 19 kb)

## Data Availability

The datasets generated and analysed during the current study are available from the corresponding author on reasonable request.

## Abbreviations

BMI: Body mass index
CT: Computed tomography
GEE: Generalized Estimating Equation
mm: Millimeters
N: Newtons
SD: Standard deviation
STROBE: Strengthening the Reporting of Observational Studies in Epidemiology
WHO: World Health Organization
